# Efferent auditory system: its effect on auditory processing

**DOI:** 10.1016/S1808-8694(15)31385-9

**Published:** 2015-10-17

**Authors:** Fernanda Acaui Ribeiro Burguetti, Renata Mota Mamede Carvallo

**Affiliations:** 1 Doctoral degree, researcher in the Laboratório de Investigação Fonoaudiológica em Audição Humana da FMUSP; 2 Livre-docência (habilitation), associate professor, Speech Therapy Course, Faculdade de Medicina da USP. Faculdade de Medicina da Universidade de São Paulo

**Keywords:** reflex, otoacoustic emissions, hearing tests, spontaneous, hearing disorders

## Abstract

Auditory processing depends on afferent and efferent auditory pathways integrity. The efferent auditory system may be assessed in humans by two non-invasive and objective methods: acoustic reflex and otoacoustic emissions suppression.

**Aim:**

Analyze the efferent auditory system activity by otoacoustic emission suppression and acoustic reflex sensitization in human subjects with auditory processing disorders.

**Method:**

Prospective study: fifty children with auditory processing disorders (study group) and thirty-eight children without auditory processing disorders (control group) were evaluated using otoacoustic emission with and without contralateral noise; and acoustic reflex with and without contralateral facilitating stimuli.

**Results:**

OAE suppression mean value was equal to or less than 1.50 dB for the control group, and equal to or less than 1.26 dB for the study group. The mean sensitization reflex value was equal to or less than 14.60 dB for the study group and equal to or less than 15.21 dB for the control group. There was no statistically significant difference between the responses from the control group and the study group in both procedures.

**Conclusion:**

The study group had lower OAE suppression values and higher acoustic reflex sensitization values when compared to the control group.

## INTRODUCTION

The hearing system is composed of integrated afferent and efferent auditory pathways. At lower levels, efferent fibers predominantly emerge from the superior olivary complex nucleus and course towards the cochlea (the efferent olivocochlear tract or the efferent medial system). Although the role of the olivocochlear bundle in hearing is unclear, certain functions have been attributed to the olivocochlear medial system: location of sound sources, auditory attention, improved auditory sensitivity, improved detection of acoustic signals in the presence of noise, and protection.^1^, ^2^, ^3^

Efferent auditory pathways may be activated by electrical and acoustic stimuli in animals;^4^, ^5^ this type of activation in humans may be done by two objective non-invasive methods: investigation of acoustic reflexes and suppression of otoacoustic emissions (OAE).^6^

Acoustic reflex threshold analysis make it possible to assess the role of efferent pathways in controlling the mechanical status of the middle ear and to collect information about brainstem auditory pathways. Acoustic reflex thresholds may be deduced from a high frequency facilitating stimulus presented prior to or simultaneously with a reflex activating tone (sensitization).^7^ The function of acoustic reflex sensitization is to improve the signal within noise in complex hearing conditions by attenuating the reflex at low frequencies.^8^

Suppression occurs when medial olivocochlear tract fibers - by outer hair synapse action - attenuate OAE responses in the presence of contralateral noise to attenuate the gain from cochlear amplification, thus decreasing cochlear membrane movement.^9^

Some authors have underlined the importance of OAE suppression when assessing the olivocochlear complex in children with auditory processing disorders, since this complex performs an important role in hearing in the presence of noise.^10^ One of the main complaints in patients with altered auditory processing is the difficulty in understanding speech in noisy environments. Studies on suppression have shown that this group of patients has decreased or absent OAE suppression, suggesting that the inhibitory effect of the efferent system is decreased.^11^, ^12^

The year 2000 Consensus Conference on the Diagnosis of Auditory Processing Disorders in School-aged Children reaffirmed the indication of electroacoustic procedures (OAE and immitance testing), as well as behavioral and electrophysiological test, to increase precision in the diagnosis of auditory processing disorders.^13^ Use of these procedures for assessment purposes may help advance knowledge about the efferent function in children with altered processing of hearing as characterized by loss of figure-background abilities and auditory closure.

The underlying hypothesis in this study was that the activity of the efferent auditory system in subjects with altered auditory processing presenting speech understanding difficulties in the presence of competing sounds may be decreased, as demonstrated by changes in OAE suppression responses and in acoustic reflex sensitization.

The purpose of this study was to verify efferent auditory system activity using OAE suppression and acoustic reflex sensitization in subjects with altered auditory processing.

## SERIES AND METHOD

This was a prospective study of 88 male and female subjects aged 9 and 10 years, seen at the Serviço de Audiologia Clínica do Centro de Docência e Pesquisa em Fonoaudiologia (Clinical Audiology Unit, Center for Teaching and Research in Speech Therapy) of our healthcare institution. The inclusion criterion for the control group (CG) was a normal auditory processing evaluation; the inclusion criterion for the study group (SG) was a diagnosis of any auditory processing disorder characterized by altered figure-background abilities and auditory closure as identified by Speech with Noise^14^ and Alternate Dichotic Dissyllable (SSW) tests.^15^ Both test, which assessed speech understanding in the presence of competing sounds, had to be altered for the purposes of this study.

Subjects had normal auditory thresholds (up to 20 dBNA at 250 to 8000 Hz), normal threshold logoaudiometry, and normal timpanometry. Two groups were generated: a control group (CG) consisting of 38 children (18 males and 20 females) and a study group (SG) consisting of 50 children (26 male and 24 female). Subjects - together with their parents or caretakers - were oriented about the aims and procedures of the study; agreement to participate required signing an informed consent form. The institution’s Research Ethics Committee approved the study (protocol n° 145/03).

A GSI 61 - Grason Stadler audiometer, Telephonics TDH 50P headphones, a Panasonic SL- S125 portable CD player and a compact disk with the assessment tests for evaluating auditory processing (elaborated by Pereira and Schochat - 1997) were used for the assessment of auditory processing. A middle ear analyzer GSI 33 - Grason Stadler version 2 was used for immitance testing, and an ILO 92 - Otodynamics version 5.61 cochlear emissions analyzer linked to an IBM microcomputer (Pentium IV CPU and colored monitor) was used for OAE emissions suppression testing.

Subjects underwent meatoscopy, pure tone audiometry, logoaudiometry, timpanometry (probe frequency = 226Hz), investigation of ipsilateral and contralateral acoustic reflex thresholds, and an assessment of auditory processing. Next, specific electroacoustic tests were done, namely OAE emissions suppression and acoustic reflex sensitization.

### OAE suppression

Tests were done with patients in a seated position on a comfortable seat within an acoustic booth. The first ear to be examined was chosen randomly, and responses were taken after adapting the probe.

**Figure 1:**
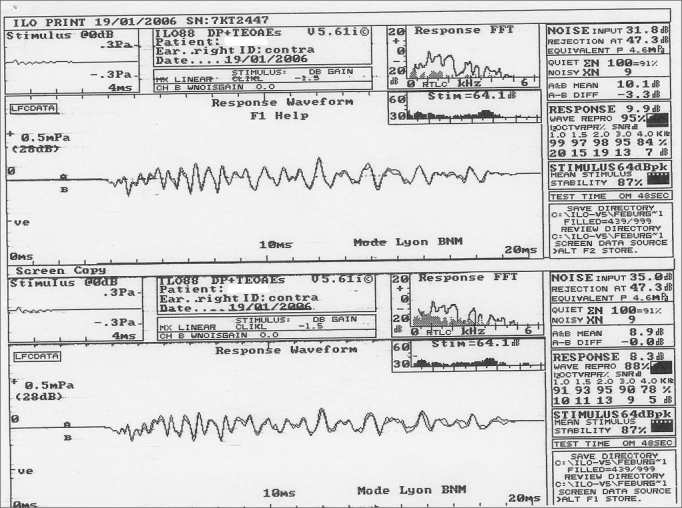
Otoacoustic emissions in the absence and presence of contralateral white noise - Source: Centro de Docência e Pesquisa - Physical Therapy, Speech Therapy and Occupational Therapy Department - FMUSP

OAE suppression testing was done using clicks as activating stimuli and white noise as suppressing stimuli, generated by the B channel of the device, and presented to the contralateral ear at 60 to 65 dB, in Lyon mode; the signal/noise ratio was between 0 and +5 dB, with a 20 millisecond window (Fig. 1).

The Lyon Mode is used to obtain automatically alternated responses with and with no noise every 20 stimuli by means of 200 linear stimuli scans. This mode was used for rapid responses with no need to adjust the probe or the equipment for noise and noiseless conditions.

Next, responses were analyzed separately with and with no competing sounds, considering the general response at intensities above background noise. Response values in the absence of noise were subtracted from the response values with noise in both groups for calculating suppression. Positive suppression values were taken into account for results analysis.

### Acoustic reflex sensitization

**Figure 2:**
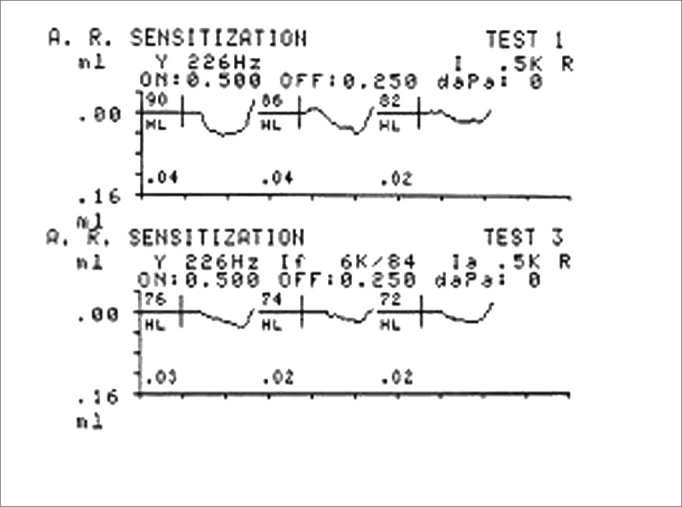
Acoustic reflex in the absence and presence of the 6 kHz facilitating stimulus - Source: Centro de Docência e Pesquisa - Physical Therapy, Speech Therapy and Occupational Therapy Department - FMUSP

Ipsilateral acoustic reflex thresholds were measured at 2 dB intervals, with no facilitating stimuli, at 500, 1000, 2000 and 4000 Hz, for acoustic reflex sensitization. Ipsilateral acoustic reflex thresholds with contralateral facilitating stimuli were then obtained (pure tone at 6000 Hz), at the same intensity at which the threshold had been measured without the facilitating stimulus (Fig. 2).

A facilitating stimulus at the maximum intensity of the device (114 dB) was presented when the acoustic reflex was absent for investigating acoustic reflex sensitization, as described above.

Ipsilateral acoustic reflex thresholds with and with no contralateral facilitating stimuli were analyzed at the end of the test. This analysis was done by comparing acoustic reflex thresholds with no facilitating stimuli subtracted from acoustic reflexes with contralateral facilitating stimuli. The analysis was done in both groups.

The Wilcoxon and the Mann-Whitney tests were used for the statistical analysis of results. The significance level for statistical inference analysis was 0.05. Statistically significant values are marked with an *, and values tending towards a difference were marked with an #.

## RESULTS

Comparative study between ears of the TOAE suppression effect

There was no statistically significant difference in the suppression effect between right ears and left ears in both groups (Table 1). There was a right ear advantage in the control group and a left ear advantage in the study group. Thus, right ear and left ear results were grouped in the subsequent analyses.

**Table 1:** Comparative analysis among OAE suppression values in the right ear (RE) and left ear (LE) in the control and study groups.

OAE suppression (in dB)	Control group		Study group	
	OD	OE	OD	OE
Mean	1,58	1,42	1,10	1,45
Median	1,60	1,10	0,75	1,20
Standard deviation	0,84	0,96	0,84	0,93
Quartile 1	0,88	0,70	0,40	0,85
Quartile 3	2,08	1,90	1,78	1,80
Size	12	13	14	11
Confidence interval	0,48	0,52	0,44	0,55
p-value	0,929		0,528	

### Comparative study among genders of the TOAE suppression effect

There was no statistically significant difference in suppression between genders in the control group. There was a statistically significant difference between genders in the study group, in which mean TOAE suppression values were higher in females (Table 2).

**Table 2:** Comparative analysis among OAE suppression values in females (F) and males (M) in the control and study groups.

OAE suppression (in dB)	Control group		Study group	
	F	M	F	M
Mean	1,48	1,54	1,57	0,85
Median	1,30	1,30	1,45	0,60
Standard deviation	0,96	0,76	0,90	0,69
Quartile 1	0,73	1,00	0,85	0,30
Quartile 3	1,98	2,10	1,88	1,15
Size	18	7	14	11
Confidence interval	0,44	0,56	0,47	0,41
p-value	0,650		0,032*	

### Comparative study among study and control groups of the TOAE suppression effect

There was no statistically significant difference between groups. Control group suppression values were higher compared to those of the study group (Table 3).

**Table 3:** Comparative analysis among OAE suppression values in the control group (CG) and the study group (SG).

OAE suppression (in dB)	CG	SG
Mean	1,50	1,26
Median	1,30	1,00
Standard deviation	0,89	0,88
Quartile 1	0,80	0,60
Quartile 3	2,00	1,80
Size	25	25
Confidence interval	0,35	0,34
p-value	0,277	

### Comparative study among ears of acoustic reflex sensitization

There was a statistically significant difference between right ears and left ears only at 2000 Hz in the control group. There was no difference in acoustic reflex sensitization between ears in the study group (Table 4). As the statistical difference was found in only one comparison, the results of both ears were grouped together for subsequent acoustic reflex sensitization analyses.

**Table 4:** Comparative analysis among acoustic reflex sensitization (ARS) values in right ears (RE) and left ears (LE) in the control and study groups.

	ARS (in dB)		Mean	Median	Standard deviation	Quartile 1	Quartile 3	Size	CI	p-value
	500 Hz	OD	13,91	10,00	13,54	5,00	15,00	23	5,54	0,756
		OE	9,91	9,00	7,70	4,00	12,00	22	3,2	
Control group	1000 Hz	OD	9,71	8,00	8,47	4,00	12,00	21	3,62	0,439
		OE	6,24	4,00	4,94	4,00	8,00	17	2,35	
	2000Hz	OD	9,44	6,00	9,20	4,00	13,00	18	4,25	0,018*
		OE	7,38	6,00	5,69	2,00	11,00	16	2,79	
	4000 Hz	OD	10,17	8,00	8,16	5,50	11,50	12	4,61	0,674
		OE	8,27	8,00	5,18	5,00	10,00	15	2,62	
	500Hz	OD	12,43	8,00	11,20	6,00	13,00	35	3,71	0,976
		OE	11,41	6,00	11,20	4,00	14,00	32	3,88	
Study group	1000Hz	OD	9,90	6,00	11,09	2,50	13,00	30	3,97	0,419
		OE	8,69	8,00	7,29	4,50	9,50	26	2,80	
	2000Hz	OD	10,93	8,00	12,37	4,00	10,00	29	4,50	0,234
		OE	8,30	8,00	6,34	4,00	10,00	27	2,39	
	4000Hz	OD	11,30	8,00	12,02	4,00	15,00	27	4,53	0,755
		OE	9,84	8,00	10,37	4,00	12,00	25	4,07	

Key: CI = confidence interval

### Comparative study among genders of acoustic reflex sensitization

There was no statistically significant difference between genders in the control group. There was a trend towards a difference only at 2000 Hz in the study group.

Mean acoustic reflex sensitization values in females were higher than those in males (Table 5).

**Table 5:** Comparative analysis among acoustic reflex sensitization (ARS) values in females (F) and males (M) in the control and study groups.

	ARS (in dB)		Mean	Median	Standard deviation	Quartile 1	Quartile 3	Size	CI	p-value
	500 Hz	F	9,18	10,00	5,83	4,00	10,00	17	2,77	0,571
		M	13,64	9,00	13,20	4,00	16,00	28	4,89	
	1000 Hz	F	7,83	7,00	4,39	4,00	10,50	12	2,48	0,464
Control group		M	8,31	5,00	8,31	4,00	10,00	26	3,19	
	2000Hz	F	6,55	4,00	5,07	4,00	7,00	11	2,99	0,423
		M	9,39	6,00	8,64	3,00	15,00	23	3,53	
	4000 Hz	F	8,44	6,00	9,53	4,00	6,00	9	6,22	0,101
		M	9,44	8,00	4,84	8,00	10,00	18	2,24	
	500 Hz	F	14,39	8,00	14,49	5,00	19,00	31	5,10	0,756
		M	9,83	10,00	6,60	4,00	12,00	36	2,15	
	1000 Hz	F	12,20	8,00	12,26	4,00	14,00	25	4,81	0,105
Study group		M	7,03	6,00	5,58	4,00	8,00	31	1,97	
	2000Hz	F	12,48	8,00	12,48	5,00	15,00	27	4,71	0,052#
		M	7,29	6,00	5,82	4,00	8,00	28	2,15	
	4000 Hz	F	13,35	8,00	15,25	4,00	15,00	23	6,23	0,413
		M	8,41	8,00	5,79	4,00	12,00	29	2,11	

Key: CI = confidence interval

### Comparative study among groups of acoustic reflex sensitization

There was no statistically significant difference between the control and study groups at all investigated frequencies. Mean acoustic reflex sensitization values were higher in the study group compared to the control group at all investigated frequencies, except at 500 Hz (Table 6).

**Table 6:** Comparative analysis among acoustic reflex sensitization (ARS) in the control group (CG) and the study group (SG).

ARS (in dB)	500Hz		1000Hz		2000Hz		4000Hz	
	SG	CG	SG	CG	SG	CG	SG	CG
Mean	11,94	11,96	9,34	8,16	9,66	8,47	10,60	9,11
Median	8,00	10,00	6,00	6,00	8,00	6,00	8,00	8,00
Standard deviation	11,13	11,14	9,45	7,24	9,94	7,71	11,17	6,59
Quartile 1	4,00	4,00	4,00	4,00	4,00	4,00	4,00	5,00
Quartile 3	14,00	14,00	10,50	10,00	10,00	13,00	12,50	10,00
Size	67	45	56	38	56	34	52	27
CI	2,66	3,25	2,48	2,30	2,60	2,59	3,04	2,49
p-value	0,979		0,732		0,534		0,963	

Key: CI = confidence interval

### DISCUSSION

This study was planned to identify differences in auditory response patterns resulting from the efferent function in children with a diagnosis of auditory processing disorders and in children with normal auditory development, based on the hypothesis that efferent auditory function is compromised in patients with altered auditory processing. Electroacoustic procedures were chosen and their responses were monitored under conditions with and with no activation of auditory efferent pathways. The fact that different responses were found for efferent pathway activation in OAE electroacoustic and acoustic reflex responses in both groups may provide clues about the functional differences associated with auditory processing disorders.

Interest in investigating variables that could differentiate electroacoustic responses in groups of children with and with no auditory processing disorders in this study was motivated by the encouraging results on OAE suppression tests in similar groups.

Acoustic reflex sensitization has been studied as a procedure for investigating medial olivocochlear tract pathways. As this method is non-invasive (similar to OAE), it becomes relevant to rethink its clinical applicability, since it may be used to evaluate efferent auditory pathways by immitance testing. Studies that have used immitance testing in subjects with auditory processing disorders have suggested that this population tends to present altered acoustic reflex thresholds; thus the importance of investigating acoustic reflex sensitization in this population group.

### Discussion about the results of TOAE suppression

There was no statistically significant difference in the results of TOAE suppression between right and left ears. Right ear values were, however, higher than left ear values in the control group, which is similar to what has been described in other studies.^16^, ^17^ TOAE suppression in the study group was higher in the left ear compared to the right ear. Lack of right ear dominance in individuals with altered auditory processing may be explained by the fact that right ear advantage is usually interpreted as reflecting left hemisphere dominance for speech and language processing and ipsilateral auditory pathway inhibition. As subjects with altered auditory processing have difficulties in dichotic conditions, dominance of left ear suppression relative to the right ear may represent a non-existence of left hemisphere dominance.^11^, ^18^

There was no statistically significant gender difference in all suppression conditions in this study. However, control group males had higher mean TOAE suppression values than females. Mean suppression values were higher in females in the study group. This may be related to the fact that males are at a higher risk for altered auditory processing and oral communication in general. A hormone-related (testosterone) hypothesis may explain a higher incidence of males with developmental conditions, possibly due to a smaller corpus callosum. The splenium (posterior portion of the corpus callosum), responsible for auditory and visual transmission between brain hemispheres, is generally wider and more bulbous in females.19 Females may thus be able to integrate visual and auditory information between both hemispheres more effectively than males, which may explain decreased OAE responses in the presence of contralateral noise.

**Chart 1:**
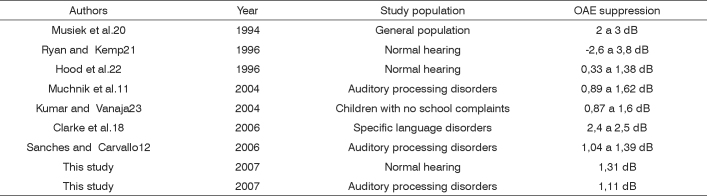
Distribution of mean OAE suppression values in various studies.

The mean suppression value was 1.50 dB in the control group and 1.26 dB in the study group. The standard deviation showed that there was not a homogeneous distribution between groups. TOAE suppression values vary, as shown in the literature (see Chart 1).

The differences among values shown in Table 7 may be explained by variations in the parameters used in each study. Thus, suppression values in each of these studies cannot be compared directly, since they were collected under different conditions and with different stimuli. Lack of homogeneity of suppression values was also found in another study,^24^ wherein the authors described that similar presented stimuli for OAEs yields different responses in different subjects, and that such intersubject variability is reflected in suppression levels.

There was no statistically significant difference when comparing suppression between the control and study groups. However, suppression values in the control group were higher compared to the study group, which suggests a decreased inhibitory effect of the efferent auditory system in children with auditory processing disorders; this findings has been reported in other studies.^11^, ^18^ Hearing, however, is a complex mechanism involving many peripheral (such as the ear) and central (such as the auditory cortex) anatomical structures. Each one of these has a specific and determinant role in hearing. Given the complexity of this system, it is impossible to attribute a single function to any structure, as the system operates as a whole, and any ability may involve more than one structure. Thus, the ability to understand speech in noise, although attributed to the efferent auditory system, surely involves other anatomical structures such as the reticular formation. Evidence suggests that when the ascending reticular activation system is stimulated the cortex becomes more alert and focused. The system thus reacts more readily to an important stimulus compared to a non-important stimulus. This may be one of the mechanisms involved in selective attention and the ability to hear in the presence of noise.^25^

All of this may explain why there was no statistically significant difference among suppression values in the groups with and with no altered auditory processing even when there were different mean responses in each group.

### Discussion of acoustic reflex sensitization results

There was no statistically significant mean difference in the comparative analysis of acoustic reflex sensitization values between right and left ears in the study group. A statistically significant difference was found between ears only at 2000 Hz in the control group wherein right ear values were higher. Another study^26^ found no statistically significant difference between ears in females with no auditory alterations; however, left ear mean values were higher at all investigated frequencies compared to the right ear.

The comparison of acoustic reflex sensitization between genders in the control group revealed that there was a statistically significant difference at 500, 1000 and 2000 Hz, with higher mean values in males. There was a trend towards a difference at 2000 Hz in the study group wherein mean values in females were higher compared to males. A study^27^ about sensitization in neonates at no risk for auditory disorders found no gender differences. Females, however, had higher sensitization means compared to males. Such gender differences may be explained by the same reasons given for OAE suppression value comparisons.

**Chart 2:**
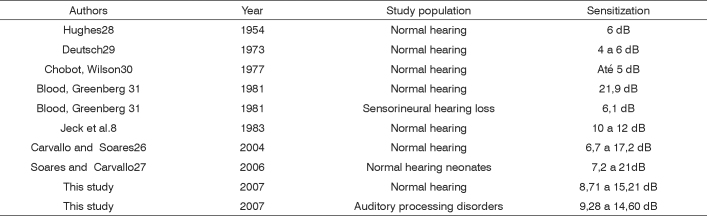
Distribution of mean acoustic reflex sensitization values in various studies.

Higher sensitization values were found at 500 Hz, with a mean 11.94 in the study group and a mean 11.96 in the control group. There is significant variability among acoustic reflex sensitization values in the literature, as shown in Chart 2.

Acoustic reflex sensitization differences among values - as with suppression - may be attributed to stimuli variations (whether ipsi or contralateral) and frequency and intensity features; there may also be differences in the samples.

There was no statistically significant difference in sensitization values between groups with and with no altered auditory processing. Study group values, however, were higher than control group values at the frequencies that were investigated (except at 500 Hz). As acoustic reflex sensitization is related with the efferent auditory system, and that one of the functions of this system is cochlear protection against intense sound, it may be inferred that this system is more effective (increased inhibitory effect) in subjects with altered auditory processing, which would interfere with speech understanding in the presence of competing sounds. Furthermore, efferent auditory pathways are activated in the presence of loud sound, altering the cochlear mechanism by means of the external hairy cells, which would decrease the traveling wave magnitude.^1^

A lower TOAE suppression effect and higher acoustic reflex sensitization in the study group may be related to the stimulus intensity used in both procedures, and consequently to the different functions of the activated efferent auditory system. The maximum suggested suppression intensity for evaluating efferent auditory function without middle ear muscle interference is 70 dB. Sensitization, however, requires obtaining an acoustic reflex threshold at a higher intensity to reach a more peripheral efferent portion. It may thus be inferred that the efferent portion evaluated by TOAE suppression is related with improved speech understanding function in noisy environments, as ambient noise occurs at an intensity similar to that used in the test. The stimulus intensity in sensitization is higher and may harm the cochlea if used for prolonged time periods. Thus it may be suggested that the efferent portion evaluated by this procedure is related with the cochlear protection function against intense sounds.

It is important to continue the investigation of objective methods for assessing subjects with auditory processing disorders, as this population group had different suppression and acoustic reflex sensitization responses.

## CONCLUSION

Mean OAE suppression values were higher in the control group compared to the study group, although this difference was not statistically significant.

Mean acoustic reflex sensitization values were higher in the study group compared to the control group, although this difference was not statistically significant.

## References

[1] Hill JC, Prasher DK, Luxon LM (1997). Evidence efferent effects on auditory afferent activity and their functional relevance. Clin Otolaryngol.

[2] Bruel MLF, Sanchez TG, Bento RF (2001). Vias auditivas eferentes e seu papel no sistema auditivo. Arq Otorrinolaringol.

[3] Hood LJ, Berlin CI, Berlin CI, Hood LJ, Ricci A. (2001). Hair cells micromechanics and otoacoustic emissions: new developments.

[4] Liberman MC, Brown MC (1986). Physiology and anatomy of single olivocochlear neurons in the cat. Hear Res.

[5] Popelar J, Valvoda J, Syka J (1999). Acoustically and electrically evoked contralateral suppression of otoacoustic emissions in guinea pigs. Hear Res.

[6] Hood LJ (1989). A review of objective methods of evaluating auditory neural pathways. Laryngoscope.

[7] Kumar A, Barman A (2002). Effect of efferent-induced changes on acoustical reflex. Int J Audiol.

[8] Jeck LT, Ruth RA, Schoeny ZG (1983). High frequency sensitization of the acoustic reflex. Ear Hear.

[9] Guinan JJ, Backus BC, Lilaonitkul Aharonson V. (2003). Medial olivocochlear efferent reflex in humans: otoacoustic emission (OAE) measurement issues and the advantages of stimulus frequency OAES. JARO.

[10] Chermak GD, Musiek FE, Chermak GD, Musiek FE (1997). Central Auditory processing disorders: new perspectives.

[11] Muchnik C, Roth DAE, Othman-Jebara R, Putter-Katz H, Shabtai EL, Hildesheimer M (2004). Reduced medial olivocochlear bundle system function in children with auditory disorders. Audiol Neurootol.

[12] Sanches SGG, Carvallo RMM (2006). Contralateral suppression of transient evoked otoacoustic in children with auditory processing disorders. Audiol Neurootol..

[13] Jerger J, Musiek FE (2000). Report Of The consensus conference on the diagnosis of auditory processing disorders in school aged children. J Am Acad Audiol.

[14] Schochat E, Pereira LP, Pereira LD, Schochat E (1997). Processamento Auditivo Central. Manual De Avaliação.

[15] Borges ACC (1986). Adaptação do teste SSW para a língua portuguesa: nota preliminar. Acta Awho..

[16] Silva CS (1997). A supressão da emissão otoacústica transiente na presença de ruído branco contralateral. [Monografia].

[17] Durante AS, Carvallo RMM (2002). Contralateral suppression of otoacoustic emission in neonates. Int J Audiol.

[18] Clarke EM, Ahmmed A, Parker D, Adams C (2006). Contralateral suppression of otoacoustic emissions in children with specific language impairment. Ear Hear.

[24] De Ceulaer GD, Yperman M, Daemers K, Driessche KV, Somers T, Offeciers FE, Govaerts PJ (2001). Contralateral suppression of transient evoked otoacoustic emissions: normative data for a clinical test setup. Otol Neurotol.

[25] Musiek FE, Oxholm VB (2000). Anatomy and physiology of the central auditory nervous system: a clinical perspective. Audiology: Diagnosis.

[26] Carvallo RMM, Soares JC (2004). Efeito do estímulo facilitador no limiar de reflexo acústico. Rev Bras Otorrinolaringol.

[27] Soares JC, Carvallo RMM (2006). Redução do limiar do reflexo acústico em neonatos sem risco auditivo. Rev Bras Otorrinolaringol.

